# The Effects of Urban Containment Policies on Public Health

**DOI:** 10.3390/ijerph17093275

**Published:** 2020-05-08

**Authors:** Jeongbae Jeon, Solhee Kim, Sung Moon Kwon

**Affiliations:** 1 Spatial Information Research Institute, Korea Land and Geospatial Informatix Corporation, 120 Giji-ro, Deokjin-gu, Jeonju-si, Jeollabuk-do 54870, Korea; jbjeon@lx.or.kr; 2 College of Agriculture and Life Sciences, Seoul National University, 1 Gwanak-ro, Gwanak-Gu, Seoul 08826, Korea; solhee1101@snu.ac.kr; 3 Division of Urban Landscape, Daegu University, Gyeongsan 38453, Korea

**Keywords:** obesity, public health, urban containment policy, greenbelt, spatial economic analysis

## Abstract

Public health risks such as obesity are influenced by numerous personal characteristics, but the local spatial structure such as an area’s built environment can also affect the obesity rate. This study analyzes and discusses how a greenbelt plan as a tool of urban containment policy has an effect on obesity. This study conducted spatial econometric regression models with five factors (13 variables) including transportation, socio-economic, public health, region, and policy factors. The relationship was analyzed between two policy effects of a greenbelt (i.e., a green buffer zone) and obesity. The variables for two policy effects of greenbelt zones are the size of the greenbelt and the inside and outside areas of the greenbelt. The results indicate that the two variables have negative effects on obesity. The results of the analyses in this study have several policy implications. Greenbelts play a role as an urban growth management policy, leading to a reduced obesity rate due to the influence of the transportation mode. In addition, greenbelts can also reduce the obesity rate because they provide recreation spaces for people.

## 1. Introduction

Policies related to obesity have focused on improving individuals’ behavior, such as eating habits and exercise. The obesity rate in Korea was 31.8% in 2018, which was an increase of 10.2% from 21.6% in 2008. Over the same period (2008–2018), the smoking rate decreased 4.4% from 26.1% to 21.7% [1]. This comparison indicates that obesity may be a greater risk to personal health than smoking, especially for adults because their obesity rate (35%) has become about three times that of younger population while the smoking rate decreased for both adults and adolescents (e.g., from 27.7% to 22.0% and from 12.8% to 6.7%, respectively). In particular, the public health sector reported that obesity is a major factor causing severe diseases that threaten personal health, such as cardiovascular disease, high blood pressure, diabetes, and respiratory disease [2].

Public health risks such as obesity are influenced by personal characteristics including genetics, diet, lifestyle, and other factors. These factors have been studied extensively in the literature. However, studies on the impact of the local spatial structure on obesity have been limited but interest is growing in the field of urban planning [3,4,5,6]. In particular, there has been considerable recent interest in the relationship between the built environment and public health. In addition, most studies have focused on the relationship between public health and urban built environments as an important aspect of micro policies (whose field-specific policy formulations or their results tend to be concerned with the behaviors or everyday decisions of citizens and, thus, are nuanced) and have examined the physical characteristics of cities such as density, centrality, land-use mix, public transportation access, and parks [5,6,7,8].

Macro policies consist of decisions that tend to be made according to top-down governmental policy formulations to bring about societal change. These include urban containment policies (UCPs) such as the establishment of urban growth boundaries, urban service areas, and greenbelt zones (greenbelt, hereafter) which have been studied over the past decade [9,10,11]. A greenbelt refers to a policy tool of spatial zoning that constrains urban sprawl and provides green environments to citizens by legally restricting development around cities and preserving nature. The primary purpose of these policies is to constrain urban sprawl. Urban sprawl causes people to mostly use individual vehicles, which reduces physical activity and leads to public health problems such as obesity. Thus, UCPs can provide an environment that can increase physical activity by managing urban growth as well as the urban characteristics to promote exercise [3]. Nevertheless, research on the relationship between UCPs and public health, particularly obesity, is still scarce.

The purpose of this study is to analyze the effects of the presence and area of greenbelts (as a policy tool of UCPs) on obesity and to derive policy implications from the main findings. While most studies have focused on the effects of greenbelts that constrain urban sprawl only, we consider the two key effects of greenbelts: constraining urban sprawl and constructing green land for citizens. This study focuses on the impact of a macro-urban policy on public health, which is distinct from other research focusing only on micro policies.

## 2. Previous Studies

### 2.1. Urban Containment Policies (UCPs) and Urban Growth

An urban containment policy (UCP) is a policy to manage urban growth by either constraining urban sprawls or promoting efficient or smart urban (inner) land uses by the varied programs or institutions of spatial zoning as well as use regulation [12]. UCPs have both advantages and disadvantages. The advantages include minimizing urbanization in rural areas, increasing urban density levels, vitalizing urban redevelopment efforts of main districts in cities, promoting the construction of new houses in urban areas, and other positive effects [9,13]. The disadvantages include increased housing and land prices, decreased inventory and quality of new dwellings due to rising construction costs, increased transportation fees with the emergence of satellite cities, as well as other negative effects [14].

This containment policy has two aspects [9]: push factors of the urban growth boundaries and pull factors of urban service areas. The push factors cause urban areas to grow as intended by limiting the urban growth boundaries and establishing greenbelts. The pull factors attract urban growth to certain target areas based on various forms of public infrastructure located along particular routes and areas, such as urban service areas. Ultimately, the main purpose of UCPs is to increase the efficiency of land use in urban areas.

“Urban growth boundaries,” which legally functions between urban and rural areas, refer to a policy measure to limit or manage urban growth. Development within boundaries causes higher density levels in urban areas due to urban growth boundaries. In contrast, outside boundaries experience lower density levels in rural areas. Urban service areas reflect a policy ensuring that growth takes place in previously planned areas to provide urban services, rather than growth in areas where urban services are not provided. Local governments can use their budgets based on the urban service area policies to provide infrastructure elements for new residents.

### 2.2. Urban Containment Policies (UCPs) and Public Health

Since UCPs generally control urban expansion and create dense cities, they increase the use of public transportation and the amount of walking for citizens. In this context, research on the relationship between urban sprawl and public health can have implications for the impact of UCPs on public health. Most studies have only focused on the relationship between urban built environments, urban sprawl, and public health [3,5,7,8,15,16,17,18]. However, few studies have been directly related to the relationship between UCPs and public health [3]. Thus, we can only infer the effects of UCPs on public health based on these published studies.

Numerous published studies on the relationship between an urban built environment and public health have focused on the effects on public health of certain physical characteristics of cities, such as residential density, mixed land use, street accessibility, and walkability. By focusing on the Atlanta metropolitan area in the United States, Frank et al. revealed that obesity can be observed based on land use patterns and car-use times by analyzing the relationship between obesity and the built environment and travel behavior [7]. Similarly, Shin et al. analyzed the relationship between obesity and commuting patterns based on a survey targeting office workers in metropolitan areas in Korea [19]. These authors argued that the obesity index of workers would decline if they used public and eco-friendly transportation. Based on the results, they reasoned that as workers spend more time in a car, their obesity index increases. Kim et al. also investigated the relationship between the proportion of obese people and the characteristics of urban environments in metropolitan areas [5]. They found that the obesity rate is affected by the bikeway range, mixed land use system, and the presence of parks in metropolitan areas. Lee et al. also identified several characteristics related to urban environments (e.g., land use, the urban form, and streets) that have a positive influence on the commuting patterns of people [8].

The relationship between obesity and urban environments can differ depending on the age group. Lee et al. studied urban characteristics affecting obesity in youths and found that youth obesity was significantly affected by mixed land use patterns [6]. However, population density, accessibility of public transportation, and urban parks were not significant variables for this group. Ewing et al. also examined the relationship between urban sprawl and body mass index (BMI), diabetes rate, and heart disease rate in 83 metropolitan areas in the United States [15]. Their results indicated that people living in densely populated cities walk considerably more, with relatively few suffering from obesity and high blood pressure. In contrast, Kelly-Schwartz et al. argued that the obesity rate has risen as urbanization progressed, based on a relationship analysis of urban sprawl and public health of 29 major metropolitan areas in the United States [16]. In addition, Aytur et al. explained that people living in metropolitan areas in the United States with powerful UCPs spend more spare time walking or biking than people without UCPs [3]. Although this relationship did not directly demonstrate that UCPs affect public health, it provides important implications about the relationships between UCPs and public health, as substantial amounts of spare time have a positive effect on individual health [20].

**Figure 1:**
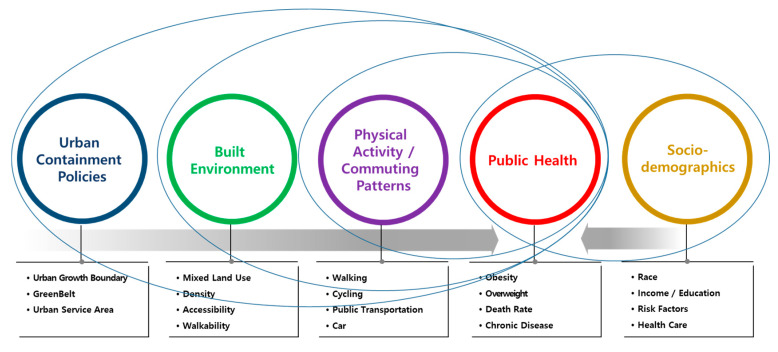
The relationship between urban containment policies and public health (Source: [3,4]).

Some studies, however, concluded that urban sprawl is not associated with public health [17,18]. Soot claimed that obesity was significantly related to the factors of population density, race, income, education, and distance from central business districts, whereas urban sprawl was not a significant variable in obesity [17]. Eid et al. also found no causal relationship between urbanization and obesity by analyzing causalities and preferences related to urban sprawl and obesity across the Unites States [18]. The relationship between UCPs and public health is illustrated in Figure 1.

UCPs increase urban built environmental factors, such as mixed land use, the density of cities, the degree of accessibility, and the level of walkability, all of which can affect physical activity as well as people’s commuting mode choices. Thus, highly dense and accessible cities increase the use of public transportation and non-motorized transportation. By increasing people’s physical activity, these public and non-motorized transportation modes have a positive effect on public health outcomes, such as obesity, an indicator of public health.

Public health outcomes are also influenced by individual socio-economic variables. Ultimately, the UCP, a macro urban policy, can have an indirect impact on public health by affecting other variables in the environment rather than directly affecting public health. Studies that have shown that urban micro and macro policies have a significant impact on public health are the bases for the basic concepts, variable selection, and analysis methodology used in this study.

## 3. Analytical Framework

### 3.1. Spatial Range

Greenbelts, a UCP push factor, drive cities to intentionally grow by limiting urban growth boundaries. The purposes of greenbelts are not only to prevent the diffusion of cities but also to provide and designate recreational green lands.

Similar to the United Kingdom, greenbelts in South Korea were designated in 14 metropolitan areas and small cities in 1971 to constrain the spread of urban areas caused by rapid urbanization and to provide a healthy living environment. Since then, greenbelts of seven small- and medium-sized urban areas were completely relieved in 1999 through the Greenbelt System Improvement Plan in South Korea. The remaining greenbelt districts are in eight metropolitan areas, namely Seoul, Busan, Daegu, Daejeon, Gwangju, Ulsan, Sejong, and Changwon. We set the spatial range in this study as counties nationwide that include greenbelts to examine the greenbelt effect on obesity. Although Jeju Island has many green areas, it was excluded from the scope of this study because it has no designated greenbelt districts.

### 3.2. Data and Variables

We considered the obesity rate (%) as a dependent variable and five factors (transportation, social, health, regional, and policy factors) that include independent variables to evaluate the effect of greenbelts on obesity (Table 1). We used national statistical data on 226 counties, including a statistical database, the Korean Household Travel Survey, and greenbelt information.

The official statistical database at the regional level is provided by the Korean Statistical Information Service (KOSIS) of the Statistics Korea. This database is provided annually at the county and local levels, focusing on 10 categories and 17 sub-categories, including population, employment, income, health, environment, and smoking (over 19 years old), drinking (monthly alcohol drinking over 19 years old), mental health, and other sub-categories. From the statistical database in 2015, we used four variables related to health, including smoking (current cigarette perceived stress over 19 years old), and the obesity rate.

The Korean Household Travel Survey provides household information, destinations, and transit modes. The household information includes attribute data of households, such as age, house type, monthly income, and vehicle ownership as well as attribute data pertaining to destinations and transit modes, such as transport modes and travel times. We used data on age, income, and number of people living in apartments from the household information and the rate of public transportation use and walking in the transit modes. For age, with 65 years or above as the reference, we divided age groups in two to evaluate the age effect: young adults (between 20 to 40 years) and middle-aged people (between 41 to 64 years).

The greenbelt information includes the status of the designation and recreation areas of each greenbelt by county as published by the Statistics Korea. Considering the policy effects of the greenbelt, the greenbelt variables can be divided into two types according to the purpose of the greenbelt: the enclosure of greenbelt and the area of greenbelt. As the first purpose of a greenbelt is to prevent urban sprawl, we can evaluate the effects of urban sprawl constraint on obesity using the first greenbelt variable (“Greenbelt Enclosure”) that dichotomizes inner-greenbelt districts and other districts in eight metropolitan areas. As the second purpose of a greenbelt is to provide green land, we can analyze the effects of green lands on obesity using the “Greenbelt Area” variable, defined as the area (1000 m^2^) whose land use is regulated under the greenbelt by county.

### 3.3. Methodology

As obesity is influenced by individual and regional spatial characteristics, an analysis with an ordinary regression model often infringes on spatial dependence and spatial heterogeneity [21]. Thus, we applied a spatial econometrics model to consider the spatial effects to prevent these statistical limitations.

The spatial econometrics model complements the shortcomings of the ordinary least square (OLS) approach and reflects the spatial effects. This spatial econometrics model is divided into three types: (1) the spatial autoregressive model (SAR) applying spatial effects to independent variables; (2) the spatial error model (SEM) reflecting spatial effects via error term; and (3) the general spatial model (SAC) reflecting spatial effects in the independent variables as well the error terms. These models are represented as Equation (1).

(1) $$Y=\rho W_{1}Y+X\beta +\varepsilon \;\varepsilon =\lambda W_{2\varepsilon }+\nu$$

Equation (1) shows that the spatial econometrics model may become the SAR when *λ* = 0, the SEM when *ρ* = 0, and the SAC when *λ* ≠ 0 and *ρ* ≠ 0. In this study, we analyze the relationships between UCPs and obesity using various regression models: OLS, SAR, SEM, and SAC types.

We tested the heteroskedasticity and nonnormality of the error term using the OLS approach before applying the spatial econometrics model. Through the Breusch–Pagan test, which verifies the heteroskedasticity of the OLS model, heteroskedasticity was found to exist in the error term, as the value from the Breusch–Pagan test was 24.32 (*p* = 0.03). In addition, through the Jarque–Bera test, which verifies the nonnormality of the error term, nonnormality was not found to exist in the error term, as the value from the Jarque–Bera test was 3.06 (*p* = 0.22).

**Figure 2:**
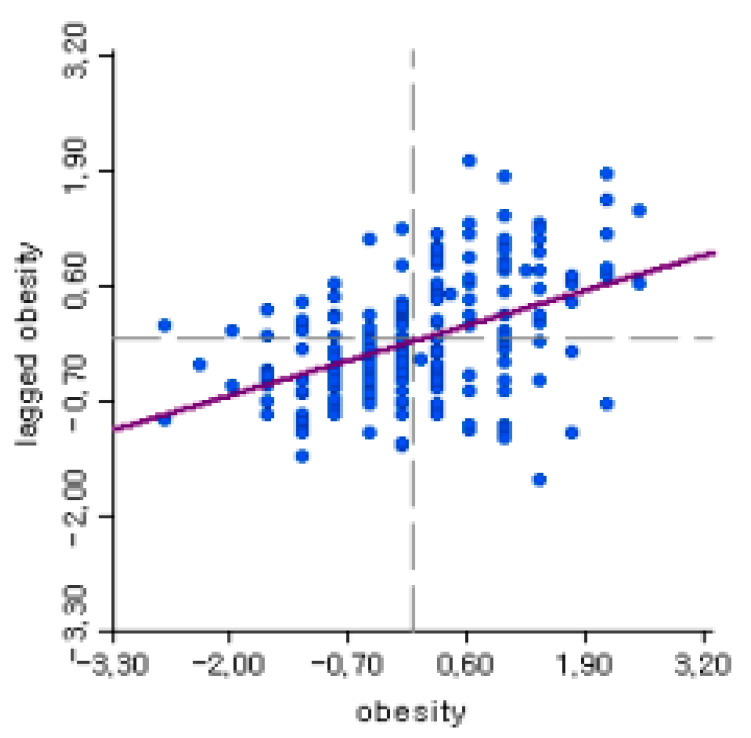
Moran’s I for obesity (Moran’s I value = 0.303).

It was also necessary to identify a spatial autocorrelation, which is an indicator of whether adjacent areas have similar or different patterns, by estimating the global Moran’s I variable. The obesity pattern/phenomenon shows a regionally spatial autocorrelation (Figure 2), and the result from Moran’s I for the obesity variable, which was 0.303 (*p* = 0.00). This result suggests that the spatial econometrics model should be utilized to explain and predict the effects of a greenbelt on obesity.

Although a test of the residuals obtained from the OLS model (Table 2), which does not consider spatial effects, showed no statistical significance in that the Moran’s I value was 0.84, apparent heteroscedasticity as well as Moran’s I for the dependent variable indicate the need for a spatial econometric model. This result suggests that the coefficients of the OLS model could still be misinterpreted when ruling out the spatial econometric models to control apparent heteroscedasticity for the analyzed areas individually having all the variables under a same spatial characteristic. Conservatively speaking, OLS and spatial econometrics models may still need to be compared with each other, as the dependent variable (obesity) appears to have locally spatial autocorrelations, which need to be examined to control or compare whether the dependents and independents are affected differently by the same spatial characteristic to make regression coefficients unbiased and consistent.

## 4. Analysis and Discussion

### 4.1. Descriptive Statistics of the Variables

**Figure 3:**
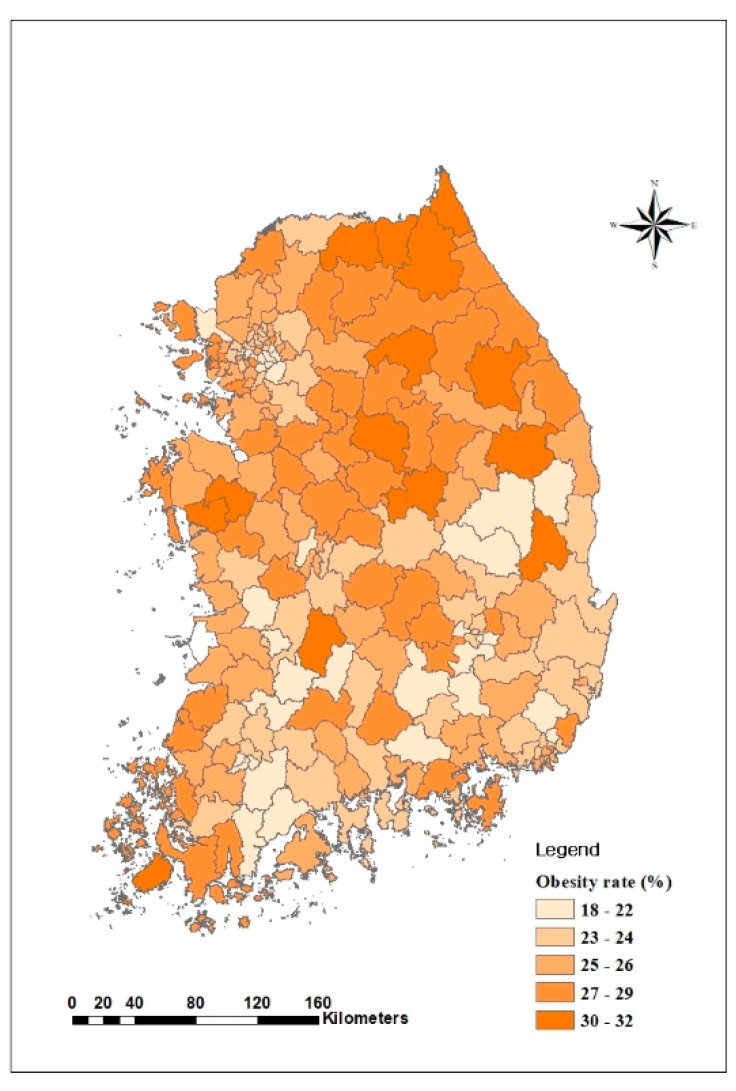
Distribution of obesity rate in Korea in 2015.

The descriptive statistics (Table 3) show that, on average, the national obesity rate was 25.3% in 2015, with the obesity rate in major metropolitan areas 0.9% lower than the national obesity rate (24.4%) and those in other areas higher than the national rate (25.8%) (Figure 3).

The transportation factors revealed a different tendency in that the usage rate of public transportation in metropolitan areas was higher than that in other areas, but the walking rate in metropolitan areas was lower than that in other areas. In particular, people in metropolitan areas used public transportation more than people who lived in other areas (23.3% in metropolitan areas, but only 8.3% in other areas), with a national average of public transportation use at 13.2% in 2015. Conversely, only 19.1% of people living in metropolitan areas often walked instead of using public transportation, whereas 30.8% of people living outside of metropolitan areas often walked instead of using public transportation.

Other factors related to rural versus urban dwellers include the residence rate, number of doctors per hundred, and average income. The average residence rate for living in an apartment was 39.2% in 2015: 50.7% for those in metropolitan areas and 33.5% for those outside of metropolitan areas. On average, one doctor treats approximately 40 citizens (i.e., 2.5 doctors per 100 citizens). The average monthly income is approximately 3.05 million KRW (in Korean won), which is between 2.0 million and 4.0 million KRW (one million KRW is equal to around one thousand USD). Rural areas accounted for 35.8% of all areas (81 rural areas), while urban areas included 145 areas.

When the greenbelt enclosure variable, a variable that distinguishes between the inside and outside of a greenbelt, is 1 for an area (county or district), it indicates that the area is enclosed by the greenbelt regardless of the total area of the greenbelt. Of the areas used in the analysis, 66 areas (29.2%) are inside the greenbelt, while 160 areas are outside of the greenbelt.

### 4.2. Effects of UCPs on Obesity

There was no spatial correlation between the residuals obtained from the OLS model, which does not consider spatial effects. Hence, the values of *ρ* and *λ* (representing spatial dependence and spatial heterogeneity) were not significant and the R^2^ values were not different in the four models. In addition, the statistical significances of all variables were not greatly different from those in the OLS model.

Most variables affecting the obesity rate were statistically significant, except for the walking variable (Table 4). Regarding the transportation factors, the greater the use of public transportation, the lower the obesity rate as well; however, the effects on obesity could not be explained by walking. Several studies revealed that people living in high-density cities use public transportation and non-motorized transportation more than individual cars [3,22,23,24]. The possible reason of this result can be inferred from the fact that people have more physical activity when they use public transportation (average is 38.9 min) because they spend longer commuting time transferring at and accessing the stops or destinations. However, walking does not have a significant impact on physical activity because the commuting time of people walking (average is 12.7 min) is short.

The significant variables pertaining to social factors were the residence rate in apartments, income, and age. The higher the residence rate in apartments and the higher the income level, the lower the obesity rate. This result indicates that people who live in apartments tend to engage in more physical activity due to legally mandatory physical facilities (such as fitness centers, tennis or basketball courts, and other activity facilities) required in apartments with more than a certain number of households [6]. Income was also negatively correlated with the obesity rate. Some researchers observed that higher incomes increase medical expenditures to improve personal health (including obesity, medical check-ups, and dietary management) [25,26].

The results for health factors show that fewer doctors per 100 people and higher drinking and smoking rates led to higher obesity rates. Since obesity can lead to other illnesses and cause increased expenses for health, higher obesity rates also lead to more medical treatments at clinics [27]. The relatively low number of doctors means that the number of hospitals in the affected regions is also low, which, in turn, results in a decrease in the opportunity to visit hospitals. Moreover, the drinking rate is positively correlated with the obesity rate because alcohol causes fat to accumulate in the body and increases food intake, in addition to the direct effects of alcohol consumption [28]. Several studies have revealed that smoking is linked to obesity-related indicators such as girth and visceral fat when controlling for personal life habits (age, exercise, drinking, and other unhealthy habits) [29,30].

The analysis also highlighted that the high percentage of elderly and inadequate infrastructure in rural areas has a relatively high effect on obesity compared to that in urban areas. The regional factors showed higher obesity rates in rural areas, likely due to the relatively higher percentage of elderly people and the lack of exercise compared to urban areas [31]. It was also reported that the rate of obesity among rural residents increased due to their lower physical activity levels, caused by low road densities, a lack of walkways, and a lack of road connectivity in rural areas [32].

Finally, the analysis shows that the presence of a greenbelt affected the obesity rate in terms of policy factors, with areas inside greenbelts having a low obesity rate. Moreover, the larger the greenbelt area in the region, the lower the obesity rate. Regarding the purpose of a greenbelt preventing urban expansion, it can increase exercise and ultimately lower the obesity rate, since a greenbelt causes cities to have higher density rates with higher usage rates of public transportation and increased walking. In addition, regarding the role of greenbelts to provide green spaces for citizens, the larger the greenbelt area, the lower the obesity rate, as the greenbelt provides citizens with leisure activities.

The result of the negative relationship between age and the obesity rate showed that lower rates of obesity are associated with “older” (e.g., 41 or above) ages. This suggests a possible connection between obesity and decreased physical activity [31]. Recently, the Korean central government’s report found [31] that younger adults tend to have higher obesity rates as they may have a nutritional imbalance from relatively little exercise despite high-fat eating habits resulting from busy everyday lives with study and work. Although “super-aged” Korean rural areas mostly lack franchised restaurants to provide unhealthy food or fitness training facilities in their lowest level of administrative area (‘ri’) with on average 150 residents while the urban corresponding one (‘dong’) has on average 2000 residents, the differences between urban and rural older people cannot be directly compared in this study. Therefore, in both the report and this study, such cultural and environmental differences like ready availability of unhealthy snack (vendors), fast food (restaurants), fitness activities, or personal training between rural and urban areas may need to be controlled and further researched. The possible interaction effects, which could also be understandable as mediating or moderating effects according to modeling, might also need to be incorporated in future research.

## 5. Conclusions

This study sought to analyze the effects of a greenbelt on the obesity rate given certain urban containment policies (UCPs). The impact of obesity rates was estimated with 13 variables grouped into five factors: transportation, socio-economic, health, regional, and policy factors. In addition, the obesity rate as a dependent variable included a spatial autocorrelation; hence, we applied the spatial econometrics models of SAR, SEM, and SAC as well as the OLS model.

For the variables affecting the obesity rate, it was found that some factors had significantly negative effects on the obesity rate, such as the rate of public transportation use, the apartment residence rate, income, the number of doctors in the area, the greenbelt area, and the areas inside the greenbelt. However, other variables of drinking, smoking, stress, and living in a rural area had significantly positive effects on the obesity rate, while older age was negatively associated with lower obesity rates.

These results provide remarkable insights into urban management planning in the transportation, socio-economic, health, and urban policy sectors. First, improvements in the public transportation infrastructure (such as the expansion of routes) can reduce obesity in rural areas based on the results of the relationship between the obesity rate and public transportation and rural areas. Second, the results demonstrating a relationship between obesity and income suggest the need for government policies to increase opportunities to provide physical activity for low-income people. Third, we can present policy direction for the health sector in terms of urban management considering the finding that a higher number of doctors per 100 people as well as healthier lifestyles lead to a lower obesity rate. Public health and leisure facilities, which can reduce stress, should be provided in cities considering the spatial identification of areas with few healthcare benefits.

Finally, the policy factor results indicated that a greenbelt is one of the major factors affecting obesity. Given that other studies have shown that greenbelts increase urban density levels, greenbelts can also serve as an urban containment policy (UCP) factor and can influence land use planning [10,11]. In addition, high density (or compact) cities increased the use of public transportation and non-motorized transportation [3,22,23,24]. Therefore, under the influence of greenbelt areas, the built environment of the city affects the use of transportation, which can ultimately play a role in lowering the obesity rate. In addition, greenbelts provide urbanites with leisure activities; thus, a larger greenbelt area can lead to a lower obesity rate. As such, it was found that greenbelts fully perform the role of a UCP as well as the role of providing leisure spaces.

Last but not least, regional differences in obesogenic factors and dietary practices also need to be further researched. In addition to the lack of physical activity, food intake will be an important dietary and obesogenic factor that may be influenced by the presence within the zone of fast food or unhealthy snack vendors and restaurants. Such zones or practices, whose data could not be obtained, need to be further considered as numerical variables to derive more viable and generalizable results.

This study is meaningful in that it analyzed the impact of greenbelts on obesity as well as on urban growth management by separating policy variables into those affecting greenbelt areas and those affecting areas inside greenbelt areas. However, it is expected that the relationship between greenbelts and obesity can be closely analyzed if certain activity data, including details about individual physical activity levels, are included and comparisons are made before and after the designation of a greenbelt zone.

## Figures and Tables

**Table 1:** Variable definitions, measurements, and expected signs.

Variables	Factor	Name	Measurement	Sign
Dependent	Public Health	Obesity	Ratio (%)	
Independent	Transportation Factors	Public Transportation	Ratio (%)	-
		Walking	Ratio (%)	-
	Social Factors	APT(Apartment)	Ratio (%)	+
		Income	10,000 KRW	-
		Age 20–40	Ratio (%)	+
		Age 41–64	Ratio (%)	+, -
	Health Factors	Doctors	Number of doctors per hundred	-
		Drinking	Ratio (%)	+
		Smoking	Ratio (%)	+
		Stress	Ratio (%)	+
	Regional Factor	Rural area	Dichotomous	+
	Policy Factors	Greenbelt Area	1000 m^2^	-
		Greenbelt Enclosure	Dichotomous	-

Note: + is the positive relationship between the obesity rate and independent variables. - is the negative relationship between the obesity rate and independent variables.

**Table 2:** The spatial autocorrelation of the residuals in ordinary least square (OLS).

Moran’s I		LM (Lagrange Multiplier) Error	LM (Lagrange Multiplier) Lag
MI (Moran’s I)	Value		
0.018	0.84	0.146	0.038

**Table 3:** Descriptive statistics of variables.

Variables		Count	Average	Standard Deviation	Max	Min
**Independent**	Obesity	226	25.32	2.70	32.00	18.00
Transportation Factors	Public Transportation	226	13.27	13.99	50.26	0
	Walking	226	26.92	14.19	90.70	7.91
Social Factors	APT	226	39.21	22.40	86.81	0
	Income	226	304.87	86.13	509.55	126.53
	Age 20–40	226	17.87	10.54	50.39	0
	Age 41–64	226	57.86	11.58	84.91	11.22
Health Factors	Doctors	226	2.48	2.28	22.04	0.83
	Drinking	226	55.74	6.33	65.90	33.80
	Smoking	226	22.57	2.74	32.80	13.90
	Stress	226	27.55	3.92	37.00	16.00
Regional Factor	Rural area	226	0.358	0.481	1	0
Policy Factors	Greenbelt Area	226	17,052.23	37,209.49	250,150.0	0.00
	Greenbelt Enclosure	226	0.292	0.456	1	0

**Table 4:** Comparison results by model: ordinary least square (OLS), spatial autoregressive model (SAR), spatial error model (SEM), and general spatial model (SAC).

Model		OLS			SAR			SEM			SAC		
Variable		Coeff.		*t*	Coeff.		z	Coeff.		z	Coeff.		z
Constant		9.472	***	3.852	9.436	***	3.935	9.863	***	4.084	9.522	***	3.95
Transportation Factors	Public Transportation	−0.043	**	−2.291	−0.043	**	−2.36	−0.045	**	−2.375	−0.044	**	−2.366
	Walking	0.002		0.124	0.002		0.111	0.002		0.116	0.001		0.098
Socio-economic Factors	APT	−0.04	***	−3.075	−0.04	***	−3.169	−0.039	***	−3.08	−0.04	***	−3.115
	Income	−0.01	**	−2.419	−0.01	**	−2.495	−0.010	**	−2.485	−0.01	**	−2.475
	Age 0–40	0.087	***	2.883	0.087	***	2.984	0.087	***	2.955	0.088	***	2.983
	Age 40–60	0.055	**	2.367	0.054	**	2.433	0.052	**	2.325	0.053	**	2.359
Health Factor	Doctors	−0.17	**	−2.381	−0.17	**	−2.454	−0.169	**	−2.442	−0.169	**	−2.437
	Drinking	0.123	***	2.867	0.122	***	2.924	0.121	***	2.862	0.121	***	2.857
	Smoking	0.22	***	3.589	0.219	***	3.696	0.217	***	3.634	0.218	***	3.654
	Stress	0.177	***	4.114	0.176	***	4.207	0.175	***	4.173	0.174	***	4.144
Regional Factor	Rural Area	1.021	*	1.919	1.024	**	1.985	1.005	*	1.947	1.025	**	1.985
Policy Factor	Greenbelt	0.000	**	−2.127	0.000	**	−2.198	0.000	**	−2.057	0.000	**	−2.132
	Inside Greenbelt	−0.951	**	−2.233	−0.949	**	−2.300	−0.900	**	−2.136	−0.92	**	−2.201
Rho (ρ)					0.005		0.196				0.011		0.396
Lambda (λ)								0.053		0.554	0.033		0.296
N		226			226			226			226		
R^2^		0.3976			0.3977			0.39851			0.3975		
Log likelihood		−487.16			−487.136			−487.05			-^1)^		
Akaike info criterion		1002.31			1004.27			1002.10			-^1)^		
Schwarz criterion		1050.20			1055.58			1049.99			-^1)^		

Notes: ^1)^ SAC did not estimate log likelihood, Akaike info criterion (AIC), and Schwarz criterion (SC) because the model used generalized method of moments (GMM). *** *p* < 0.01, ** *p* < 0.05, * *p* < 0.1. t is t-value, z is z-value.
